# Differential discontinuation by covert use status in Kenya^☆^

**DOI:** 10.1016/j.conx.2023.100102

**Published:** 2023-10-16

**Authors:** Dana Sarnak, Shannon N. Wood, Phil Anglewicz, Elizabeth Gummerson, Peter Gichangi, Mary Thiongo, Caroline Moreau

**Affiliations:** a Department of Population Family and Reproductive Health, Johns Hopkins Bloomberg School of Public Health, Baltimore, MD, United States; b International Centre for Reproductive Health Kenya, Nairobi, Kenya; c Department of Public Health and Primary Care, Ghent University Faculty of Medicine and Health Sciences, Ghent, Belgium; d Soins et Santé Primaire, CESP Centre for Research in Epidemiology and Population Health U1018, Inserm, Villejuif, France

**Keywords:** Covert contraceptive use, Discontinuation of contraception, Family planning, Longitudinal data, Partner dynamics, Sub-Saharan Africa

## Abstract

**Objectives:**

Qualitative research suggests that covert users may be more likely to discontinue contraception due to the logistics of discretion and fear of disclosure. This study sought to quantify whether covert users are more likely to discontinue contraception than overt users.

**Study design:**

We used a national longitudinal survey from Kenya conducted from November 2019/February 2020 to November 2020/April 2021 to test whether the time to discontinuation between covert and overt users still in need of contraception differed using survival analyses over a period of 5 years since method initiation.

**Results:**

Multivariate Cox regression results showed there was an interaction with time and covert use on the risk of discontinuation; for every additional month of use, there was an increased risk of discontinuation of covert users compared to overt users (3% increased hazard, *p* = 0.02). At 1 and 2 years, there were no differences in the hazard of discontinuation (adjusted hazard ratio [aHR]_1 year_ 0.95, 95% CI 0.54–1.65 and aHR_2 years_ 1.37, 95% CI 0.85–2.21), yet at 3, 4, and 5 years, the hazard of discontinuation was higher for covert compared to overt users (aHR_3 years_ 1.99, 95% 1.11–3.56; aHR_4 years_ 2.89, 95% CI 2.0–6.40; aHR_5 years_ 4.18, 95% CI 1.45–12.0).

**Conclusions:**

These results suggest efforts are needed to support covert users in managing their contraceptive use and for improving contraceptive counseling surrounding covert use. Our findings shed light on the increasing challenge covert users face after approximately the first 2 years of use; covert users require additional follow-up in both research and care provision.

**Implications:**

Covert users are at a higher risk of discontinuation of contraception while still trying to avoid pregnancy, particularly after the first 2 years of use. Family planning providers and programs must protect access to and maintain the privacy of reproductive services to this population, focusing on follow-up care provision and counseling.

## 1 Introduction

Covert use of contraception, which refers to the use of contraception without the knowledge of one’s partner or other family members, is a strategy women use to challenge opposition to family planning without suffering the consequences. Covert use is notoriously hard to measure, given the sensitive nature of the behavior and potential consequences of disclosure. Yet, recent studies have estimated the prevalence of the behavior in sub-Saharan Africa (SSA) countries to be around 10% [1], highlighting this practice as a common strategy across diverse contraceptive landscapes.

While qualitative research in SSA discusses the social and economic gains of using contraception covertly, the need to conceal the use of contraception can also come at a cost, including potential consequences of discovery. Covert users are at a disadvantage for several reproductive outcomes; specifically, they may be more likely to stop using their method and may be less inclined to seek treatment or switch methods due to feared or experienced side effects, especially if side effects (e.g., menstrual changes) expose their use [2], [3], [4]. Using contraception covertly can also be logistically challenging: a qualitative study in Senegal showed that covert users of pills and injectables were late in obtaining their method because they often had to wait until their husbands were traveling to attend the clinic; seek services at a distant facility to avoid being identified; or pretend to be sick as an excuse to go to a facility [5]. For women experiencing reproductive coercion, using contraception covertly may also be dangerous—in Nairobi, women described cyclic experiences of reproductive coercion and covert use, where multiple attempts were needed to secure a method that their husbands would not interfere with [6].

Ensuring continuity of contraceptive method use is critical to meet the needs of women and girls who are seeking to avert pregnancy [7]. To date, only one published quantitative study that the authors are aware of has compared contraceptive use dynamics between covert and overt users, finding no significant differences in the risk of discontinuation or switching [8]. However, this pilot study conducted in Uganda was limited in sample size, suffered relatively high rates of attrition, and used logistic and multinomial regressions to calculate differences in discontinuation; therefore, there is a need for further longitudinal investigation. The aim of this study was to compare time to discontinuation between covert and overt users in Kenya.

## 2 Methods

We used data from a nationally representative longitudinal survey conducted in Kenya as part of the Performance Monitoring for Action Project [9]. A multistage stratified cluster design was used to draw a probability sample of households and females of childbearing age (15–49 years). Women who met this eligibility and who were usual members of the household or who slept in the household the night before were approached for interview. After providing informed consent, 9478 completed a baseline questionnaire in phase 1 (P1) from November 2019 to February 2020. Of these women, 8729 women aged 15 to 48 years (e.g., who did not age out) consented to participate in future surveys. Of those, 6935 women were relocated 1 year later in phase 2 (P2), reflecting a retention rate of 79.4%. Women who were lost to follow-up at P2 (*n* = 2504) were younger, had lower parity, higher education levels, higher wealth status, more likely to be urban, not married, less likely to be using contraception, and more likely to be covert users. However, inverse probability weights (described below) were used in the analysis to preserve the original P1 sociodemographic composition (Appendix 1).

Interviews were conducted by trained local female interviewers who collected information about women’s sociodemographic backgrounds, their fertility history, fertility intentions, and contraceptive behaviors. P2 also included a retrospective reproductive calendar, in which women were asked to recount pregnancies and contraceptive use for each month between P1 and P2, as well as reasons for discontinuation any time they stopped a method. More information about the Performance Monitoring for Action study design and survey instrument can be found elsewhere [10] and https://www.pmadata.org/data/survey-methodology.

### 2.1 Analytic sample

**Fig. 1:**
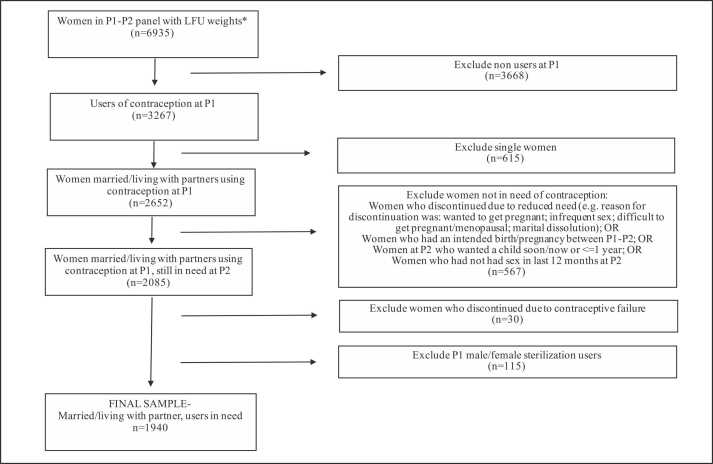
Flow diagram depicting inclusion/exclusion criteria for the study sample, Kenya 2019–2020. *Notes: LFU, loss to follow-up. LFU weights were created for women who consented to be followed up at P1, did not age out at P1 (>49 y), spent the night before in the household (de jure) at both P1 and P2, and completed both surveys. P1, phase 1, P2, phase 2.

In this research, we were interested in examining cumulative discontinuation of contraception among partnered contraceptive users who remained in need of contraception over the observation period; in other words, among self-reported fecund and sexually active women who wished to avoid pregnancy. To achieve this, we implemented several inclusion/exclusion criteria. First, our sample was limited to women who were married or living with a partner and were using contraception at P1. Second, in order to study discontinuation rates for method-related reasons as opposed to contraceptive discontinuation because women were no longer in need of contraception (no sexual activity, had an intended pregnancy, or intended to become pregnant), we excluded women who were not sexually active in the last 12 months (at either P1 or P2) or who reported their reason for discontinuation of contraception as due to reduced need (e.g., discontinuation due to desire to become pregnant, infrequent sex, marital dissolution, perceived infecundity). We excluded women who discontinued and had a subsequent pregnancy that was reported as being intended. We also excluded women whose discontinuation was due to a contraceptive failure, for example, became pregnant while using contraceptive either due to a method-related (i.e., failure of a method to work as expected) or user-related (i.e., from incorrect or inconsistent use of a method) failure [11]. Furthermore, we excluded women who reported at P2 that they wanted a child within the next year. Finally, we excluded women who reported their method was female sterilization at P1, given the permanence of this method. Our study sample comprised 1940 women (see Fig. 1).

### 2.2 Measures

Our outcome measure was time to discontinuation. Total duration of contraceptive use was constructed by adding the total months of use reported at the P1 survey (e.g., the months between initiation [T0] and the P1 survey) to the months of use reported in the P2 retrospective calendar between P1 and P2. Because our sample consisted of users at P1, women who discontinued between surveys were characterized as discontinuers. Those who continued using over the study period and were still using at P2 were considered continuers and were right censored. Additionally, women who discontinued their P1 method but switched methods within 3 months of discontinuation were also classified as continuers until subsequent discontinuation or censoring at P2 survey. This decision was made because the number of women who switched methods was too small to analyze as a separate category (*n* = 155), and it did not differ between covert and overt users; 8% of covert users switched compared to 9% of overt users (*p* = 0.56). We allowed women a 3-month period to switch methods based on prior research [12] and given the potential coverage of methods, such as the injectable.

Our exposure variable of interest was covert or overt use, which we defined using responses to the question: “Does your partner/husband know that you are using [method]?” The question was asked of all female-controlled contraceptive users (implants; intrauterine devices; injectables; pill; emergency contraception; female condom, standard days/cycle beads, rhythm; other traditional). Women who responded “no” were considered covert users, while women who responded “yes” were defined as overt users. Women who were using male-dependent methods (male condoms or withdrawal) were not asked the question, as their partners were directly involved and were classified as overt users [1], [13], [14].

We considered the following sociodemographic covariates shown to be associated with covert use in previous research [1], [13], [14] and categorized them into the following: age (15–24 years, 25–34 years, and 35+ years), parity (0–2 children ever born, 3–4 children ever born, and 5+ children ever born), highest education level achieved (none/primary, secondary+), household residence (urban/rural), and household wealth tertile (lowest, middle, highest). Furthermore, we distinguished between the use of long-acting reversible contraceptive (LARC) methods (i.e., intrauterine devices and implants) and shorter-acting methods (SAMs), such as injectables and pills, because method choice likely differs between covert and overt users and because discontinuation rates differ substantially between LARCs and SAMs.

### 2.3 Methodological approach

In our analysis, we first calculated P1 descriptive statistics of our sample and compared these for covert and overt users using design-based F-statistics. In exploratory analyses of the 1940 contraceptive users in our study sample, we found that women tended to be longer-term users with a mean duration of use prior to P1 of 31 months and median of 21 months (interquartile range 19 months to 52 months); this is not surprising, given that the inclusion criteria for this study included being in need of contraception between P1 and P2 (e.g., wanting to space or limit childbearing, being sexually active and in a partnership, etc., as described above). Therefore, we decided to look at contraceptive use dynamics over a potential observation period of 60 months. We compared crude 12-, 24-, 36-, 48-, and 60-month discontinuation rates (defined as number of discontinuations/person-months) between covert and overt users using survival analysis. While 12-month discontinuation rates are often used in the field, we opted to look at a longer observation period, given the fact that many users entered at P1 as long-term users. Additionally, to account for women who had been using prior to entrance into the study at P1, we utilized delayed entry methods in our survival analyses to account for use time prior to P1 [15], as done in research on discontinuation with similar study design to ours [16]. This approach led to the exclusion of another 297 women who had been using for more than 5 years at baseline due to these women never entering the risk pool given our observation period of 5 years; this exclusion results in an analytical sample of 1643 women. Finally, given the fact that we may be underrepresenting short-term users, we quantified the number of women who adopted and discontinued between P1 and P2 (e.g., short-term users that we may be “missing”) and subsequently overweighted the short-term users (using for ≤12 months) in our study sample by this factor and underweighted long-term users (using for >12 months) by the inverse of that factor.

We conducted crude and adjusted multivariable Cox regressions predicting the hazard of discontinuation by type of use, adjusting for sociodemographic covariates and method type (SAMs vs LARCs). In exploratory analyses, log-log survival plots and Schoenfeld residual-based test for proportional hazards revealed that the proportional hazard assumption for the full duration of time (60 months) was violated. To account for nonproportional hazards, we introduced a time-varying covariate for the type of user in the model, in other words, an interaction of the variable with time. We also explored potential interactions between time to discontinuation and covert/overt use and method type (SAM vs LARC).

A secondary analysis of this study was to examine whether covert use status changed between P1 and P2. We were only able to test this among the subset of women who were classified as continuers (still using at P2). We calculated the percent of P1 overt users who continued using overtly or had transitioned to covert use. Likewise, we calculated the percent of P1 covert users who remained covert users and who had transitioned to overt use.

### 2.4 Sensitivity analyses

We ran several sensitivity analyses to check the robustness of our regression results. We ran a sensitivity analysis excluding users of male condoms and withdrawal methods from the overt user group, given that some studies on covert use exclude these male-controlled methods. We also repeated analyses excluding women classified as switchers to see if our results held.

All analyses were weighted for the complex survey design. In addition, we used weights computed in the parent study to account for the likelihood of differential loss to follow up from P1 to P2 using inverse probability weighting methods [17]. These were created by estimating a logit model on the probability that a woman who participated in P1 was surveyed at P2 using age, marital status, parity, education, wealth, and urban/rural residence as regressors. The resulting attrition weight was the inverse of this predicted probability. The final survey weight preserves the P1 sociodemographic characteristics; this can be seen by comparing columns 2 and 4 in Appendix 1. All analyses were conducted in STATA 17 (StataCorp, College Station, TX).

## 3 Results

Table 1 presents the sociodemographic characteristics by covert and overt users. Approximately 11% of contraceptive users reported using their method covertly. Covert users tended to be older, have attained lower levels of school, have more children, and be in lower wealth tertiles than overt users. Method mix differed between overt and covert users; specifically, a higher percentage of overt users were implants compared to more covert users using injectables.

**Table 1:** Weighted percentage distributions of characteristics of contraceptive users at phase 1 in Kenya (*N* = 1643), 2019

Descriptive characteristics at P1		Total	Overt	Covert	*p* value
Individual characteristics					
Age (y)	15–24	20	20	15	
	25–34	48	49	43	
	35+	32	31	42	0.05
Highest schooling level	None/primary	56	55	65	
	Secondary+	44	45	35	0.05
Parity	0–2 children	35	36	25	
	3–4 children	41	41	37	
	5+ children	24	22	39	<0.01
Household characteristics					
Household wealth	Lower	35	34	50	
	Middle	35	36	25	
	Highest	30	31	25	<0.01
Residence	Rural	68	68	72	
	Urban	32	32	28	0.36
Contraceptive characteristics					
Method mix					
	Implant	46	47	39	
	IUD	4	4	3	
	Injectable	35	33	47	
	Pill	7	7	4	
	Condoms	2	2	0	
	Other modern	3	3	4	
	Traditional	4	4	3	0.08
Method type	Short-acting method	50	49	58	
	LARC	50	51	42	0.13
Duration of use prior to P1	Mean (months)	19	19	19	0.95
	Median (months)	16	16	16	
Percent of users			89	11	
					
Total (N)		1643	1470	173	

IUD, intrauterine device; LARC, long-acting reversible contraceptive.

**Fig. 2:**
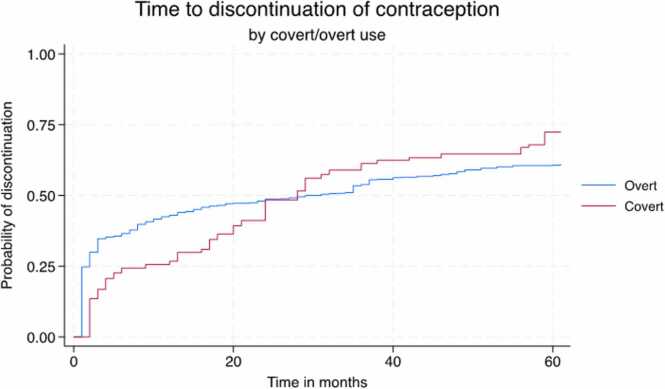
Cumulative incidence of discontinuation of contraception among Kenyan users by overt/covert use status (*N* = 1643), 2019–2020. Notes: Appendix 2 shows the 12-, 24-, 36-, 48-, and 60-mo discontinuation rates among all women by overt/covert use status.

Figure 2 presents the Kaplan-Meir curves for time to discontinuation between covert and overt users. Discontinuation rates at 12 months were lower among covert users compared to overt users (27% vs 43%, respectively), but by 24 months, they were almost equal (48% vs 49%, respectively). At 36, 48, and 60 months, covert users had higher rates of discontinuation than their overt counterparts (61% vs 54%, 65% vs 58%, and 72% vs 61%, respectively).

Our Cox regression results also reflect a dynamic story. Table 2 presents the results for the full regression model predicting the hazard of discontinuation by type of use, adjusting for sociodemographic covariates, method type, and a time-varying covariate for the type of user × time. While the main effect of covert use on time to discontinuation shows a reduced risk of discontinuation (adjusted hazard ratio [aHR] 0.66, 95% CI 0.31–1.40), there is a significant interaction with time such that for every additional month, there is an increased risk of discontinuation of covert users compared to overt users (aHR for interaction 1.03, 95% CI 1.01–1.06, *p*-value = 0.018). Table 3 presents how the aHRs for covert/overt use vary over time; at 1 and 2 years, there are no statistically significant differences in the hazard of discontinuation between covert and overt users (aHR 0.95, 95% CI 0.54–1.65 and aHR 1.37, 95% CI 0.85–2.21, respectively), yet at 3, 4, and 5 years, the hazard of discontinuation is higher for covert users compared to overt users (aHR 1.99, 95% 1.11–3.56; aHR 2.89, 95% CI 2.0–6.40; aHR 4.18, 95% CI 1.45–12.0). The results also indicate LARC users had lower risk of discontinuation compared to SAM users (aHR 0.31, 95% CI 0.21–0.45). Models with interactions between user type and method type were run; the interaction term was not significant. Similar findings were found in sensitivity analyses limited to female-controlled methods and excluding switchers (Appendix A, Appendix A).

**Table 2:** Adjusted hazard ratios for hazard of discontinuation of contraception in Kenya (*N* = 1643), 2019–2020

Characteristic		Adjusted HR			
		Adjusted HR	Lower CI	Upper CI	*p*-value
Age (y)	15–24 (ref)				
	25–34	1.14	0.68	1.91	0.62
	35+	1.58	0.80	3.13	0.19
Highest schooling level	None/primary (ref)				
	Secondary+	0.91	0.63	1.31	0.63
Parity	0–2 children (ref)				
	3–4 children	0.76	0.48	1.22	0.26
	5 plus children	0.84	0.45	1.57	0.59
Household wealth	Lower (ref)				
	Middle	0.64	0.42	0.98	0.04
	Highest	0.81	0.49	1.35	0.42
Residence	Rural (ref)				
	Urban	1.15	0.77	1.71	0.49
Method type	Short-acting method (ref)				
	Long-acting method	0.31	0.21	0.45	<0.01
Type of use	Overt use (ref)				
	Covert use	0.66	0.31	1.40	0.27
Interaction	Time (months) × type of use	1.03	1.01	1.06	0.02

HR, hazard ratio.

**Table 3:** Adjusted hazard ratios for hazard of discontinuation of contraception comparing covert users vs overt users by month in Kenya (*N* = 1643), 2019–2020

Month since discontinuation		Adjusted HR	Lower CI	Upper CI	*p*-value
Month	12	0.95	0.54	1.65	0.85
	24	1.37	0.85	2.22	0.19
	36	1.99	1.11	3.56	0.02
	48	2.88	1.3	6.4	0.01
	60	4.18	1.45	12.03	0.01

HR, hazard ratio.

*Adjusted for age, education, parity, household wealth, residence, and method type.

Table 4 presents the change in covert/overt use status among the 1515 women classified as continuers. Among P1 overt users, almost all (96%) remained overt users 1 year later. Among P1 covert users, 50% reported using overtly at P2.

**Table 4:** Change in overt/covert use status from P1 to P2 among continued users in Kenya (*N* = 1515), 2019–2020

Overt/covert use status	P2 overt users, *n* (row percent)	P2 covert users, *n* (row percent)	*n* (row percent)	*p*-value
P1 overt users	1317 (96.4)	50 (3.7)	1367 (100)	
P1 covert users	81 (50.0)	67 (50.0)	148 (100)	<0.01

*Restricted to women who were defined as "continuers" because women at P2 were only asked covert/overt status if they were using (*n* = 1515).

## 4 Discussion

Our study used longitudinal survey and retrospective calendar data to quantify differential contraceptive use patterns between covert and overt users in need of contraception. While we had hypothesized that covert users would be at an increased risk of discontinuation compared to overt users, our results suggest a more nuanced and dynamic story. Somewhat surprisingly, we found that over approximately the first 2 years of use, there were no differences in time to discontinuation between covert and overt users. However, after the first 2 years of use, covert users had an increased hazard of discontinuation compared to overt users, a pattern that persisted until the end of the 5-year observation period. Of note, while most of the overt users remained overt users between P1 and P2, 50% of P1 covert users had transitioned to overt use by P2.

While it is encouraging that there were no differences in the hazard of discontinuation between covert and overt users for the first 2 years, there seems to be an increased or compounded burden of continuation for them after 2 years. The qualitative literature has highlighted the plethora of obstacles covert users face when constrained by secrecy. Such hindrances include access barriers, including difficulties in making appointments, traveling to facilities, requirement of spousal consent for family planning provision, stockout issues, and cost [4], [5], [18], [19]. Covert users may also face increased challenges around continuation due to managing menstrual side effects, including increased bleeding or decreased libido that may risk exposure of covert use [2], [3], [4], [18]. The increased emotional burden on women may lead some to prematurely discontinue; numerous qualitative studies have shown that covert use can cause internal conflict for a woman, given the deception it creates within the partnership [20], [21], opposition with her religion [3], [21], [22], and stigma among other women [21]. Finally, covert users who are successful in preventing pregnancy may face increased suspicion from their partners around covert use of contraception or infertility; notably, such suspicions that would only increase over time with lack of conception. While the motivation to avoid pregnancy and conceal use may outweigh these pressures early on, dealing with any one of these issues, as well as the combination, may become insurmountable for the covert user over time.

Higher discontinuation for covert users after longer periods may also be rooted in partner suspicions and, in worse cases, reproductive coercion and/or intimate partner violence. Specifically, for women experiencing reproductive coercion and using covertly, partner suspicion may mount when they do not become pregnant, leading to increased susceptibility for subsequent reproductive coercion [6], [23]. Unfortunately, the more successful women are in covert use, the more they may experience the coercion tactics from a partner, ranging threats surrounding resources and housing, child custody, and potentially violence (including forced sex), all of which could lead to discontinuation.

These results have clinical implications. First, they call for greater efforts during initial contraceptive counseling to understand women’s circumstances when selecting a method. The dominant methods that women are using in Kenya—implants and injectables—may seem ideal for covert use from a provider perspective, given the ease of concealment [19], but in turn, is known for producing contraceptive-induced menstrual bleeding changes. These contraceptive-induced menstrual bleeding changes have been linked to dissatisfaction with and discontinuation of methods [24]; covert users face the additional, and potentially serious, problem of contraceptive-induced menstrual bleeding changes leading to exposure of use. Other literature has also highlighted how providers may dismiss client concerns about side effects as “myths and misconceptions” [19]; yet, these concerns around menstrual bleeding are highly warranted for women attempting to conceal their contraceptive use. Therefore, to provide patient-centered quality care, we recommend that providers attempt to gauge how problematic contraceptive-induced menstrual bleeding changes are to a woman during contraceptive counseling and method provision, which may lead to the adoption of a less effective or shorter-acting method that would be better suited for discreet use.

Furthermore, our results highlight the particular challenge covert users face in continuing beyond the first 2 years of use. Therefore, providers should consider continual follow-up with these women to help maintain or switch methods over longer-term periods, for example, to facilitate switching if side effects are threatening exposure. Continued follow-up on method use is especially imperative for those women experiencing reproductive coercion and/or intimate partner violence to ensure that women are not only able to avert unwanted pregnancy but also remain safe. More research is needed on the specific components of interventions that may help achieve this, given that much of the literature evaluating the effects of counseling on discontinuation (which has found mixed results) has been focused on short-term follow-up periods [25], [26]. Strategies that have been proposed specifically to facilitate covert use continuation include embedding family planning provision into other points of care, such as maternal and child health care services [3], [27]. Finally, there is a small but growing body of work testing and studying innovative contraceptive technologies that are designed for discreet use [28], [29], which could further aid this specific population in continuation.

While our study design precludes the ability to test whether the transition from covert to overt use was associated with discontinuation, our results suggest that a common trajectory of covert users who continue use appears to be shifting to overt use. This has been referenced in qualitative work; men and women in SSA contexts have recognized that in some instances, disclosure of covert use can lead to improved spousal communication around family planning and overt use [4]. This can be the case when there is an initial lack of spousal discussion around family planning and fertility intentions; women who do not know their partner's fertility preferences are more likely to use covertly [30]. The transition from covert to overt use deserves further investigation around the factors that facilitate this negotiation. This finding reiterates the importance programs and environments that facilitate and encourage overt use of contraception where feasible and safe.

Our study has several limitations. First, the use of the direct question to measure covert use likely underestimates the true prevalence [31]; given the sensitive nature of the behavior, women who use contraception covertly may similarly be reluctant to report use in a survey. Second, we collected data on covert use at the time of the P1 and P2 surveys; however, the measure of time to discontinuation included use time prior to the P1 survey, on which we do not have information on covert status. Third, our analytic sample of women is skewed to long-term users due to the inclusion criteria of being in need at P1 and P2, and therefore, we are missing both covert and overt users who adopted and discontinued quickly (e.g., coital dependent method users such as emergency contraception) and their discontinuation rates may be systematically different. However, our delayed entry approach and overweighting of short-term users in our sample attempt to attenuate any lead-time bias. Fourth, there is a growing body of work on the overall limitations of the contraceptive calendar due to recall bias [32], [33], [34], [35]. Recent evidence has shed light on inconsistencies in calendar reporting among covert users specifically [36]. We acknowledge that this is an unavoidable limitation of the data. Fifth, we recognize that contraceptive discontinuation is an imperfect measure that does not always consider women’s choice in discontinuing. While our goal was to focus on women who discontinued while still desiring to avoid pregnancy, there may be marked differences in women who are in need of contraception vs those who want to use contraceptive methods [16], [37]. Pregnancy intentions and desires to use contraception are constantly changing, and despite our efforts to hone in on a population desiring to avoid pregnancy over an observation period, these needs are in constant flux, and therefore, measurements of them are unavoidably crude. Future research should aim to examine more innovative measures for maximizing women’s choice in using contraception and discontinuing use [38].

Despite these limitations, to our knowledge, this study is the first to examine differential discontinuation between covert and overt users among women in need of contraception in low-resource setting. Our study provides empirical evidence to what has been shown in the qualitative literature—covert users have more difficulty in continuing methods over a longer-term period of attempting to prevent pregnancy. Given that discontinuation differences may not be readily apparent in the short-term, covert users require additional follow-up in both research and care provision.

## Data statement

The data sets analyzed in this study are available in the Performance Monitoring for Action repository, https://www.pmadata.org/, and can be obtained for free by requesting access online.

## Declaration of Competing Interest

None.
