# Supplementary material for: Differential discontinuation by covert use status in Kenya
Source: Contracept X. 2023 Oct 16;5:100102. doi: 10.1016/j.conx.2023.100102 (PMC10625140; doi:10.1016/j.conx.2023.100102)
Supplement: Supplementary file 1 — Supplementary material [file mmc1.docx]

**Appendix 1. PMA Kenya full sample characteristics, with P1 weights, LFU weights**

|  |  | **PMA KENYA FULL SAMPLE** | | | | |
| --- | --- | --- | --- | --- | --- | --- |
|  |  | P1 full sample, with P1 weights | P1-P2 panel sample, with P1 weights | P2 lost to follow up, with P1 weights |  | P1-P2 panel sample with inverse probability LFU weights |
| Age (mean) |  | 28.8 | 29.4 | 27.0 |  | 28.7 |
| Education | None/Primary | 49.7 | 52.6 | 41.3 |  | 49.2 |
|  | Secondary+ | 50.3 | 47.4 | 58.7 |  | 50.8 |
| Parity (mean) |  | 2.4 | 2.6 | 1.7 |  | 2.4 |
| Household wealth tertile | Lower | 35.6 | 37.8 | 29.0 |  | 35.4 |
|  | Middle | 34.6 | 36.2 | 29.8 |  | 34.7 |
|  | Highest | 29.9 | 26.0 | 41.2 |  | 30.0 |
| Residence | Urban | 30.2 | 26.4 | 41.1 |  | 30.5 |
|  | Rural | 69.8 | 73.6 | 59.0 |  | 69.5 |
| Marital status | Not married | 40.9 | 36.4 | 53.4 |  | 41.2 |
|  | Married/living with partner | 59.2 | 63.6 | 46.6 |  | 58.8 |
| CPR |  | 45.7 | 47.4 | 40.8 |  | 45.7 |
| Covert use |  | 15.1 | 14.0 | 19.0 |  | 14.2 |
| N |  | 9478 | 6935 | 2504 |  | 6935 |

**Appendix 2. 12-, 24-, 36-, 48-, and 60-month discontinuation rates among all women, by overt/covert use status, Kenya**

|  | Type of user | | TOTAL |
| --- | --- | --- | --- |
| Discontinuation rate at: | Overt use | Covert use |  |
| **12 months** | 0.4297 | 0.2672 | 0.4048 |
| **24 months** | 0.4870 | 0.4835 | 0.4745 |
| **36 months** | 0.5387 | 0.6129 | 0.5348 |
| **48 months** | 0.5845 | 0.6464 | 0.5804 |
| **60 months** | 0.6075 | 0.7240 | 0.6139 |

**Appendix 3. Adjusted hazard ratios for hazard of discontinuation of contraception in Kenya (2019-2020), among female-controlled method users**

|  |  | ADJUSTED HAZARD RATIO | | | |
| --- | --- | --- | --- | --- | --- |
|  |  | Adjusted HR | Lower CI | Upper CI | p-value |
| Age, years | 15-24 (ref) |  |  |  |  |
|  | 25-34 | 1.35 | 0.79 | 2.29 | 0.37 |
|  | 35+ | 1.95 | 0.97 | 3.94 | 0.06 |
| Highest schooling level | None/Primary (ref) |  |  |  |  |
|  | Secondary+ | 0.85 | 0.58 | 1.25 | 0.41 |
| Parity | 0-2 children (ref) |  |  |  |  |
|  | 3-4 children | 0.71 | 0.44 | 1.16 | 0.26 |
|  | 5 plus children | 0.76 | 0.40 | 1.44 | 0.40 |
| Household wealth | Lower (ref) |  |  |  |  |
|  | Middle | 0.61 | 0.39 | 0.96 | 0.03 |
|  | Highest | 0.80 | 0.47 | 1.38 | 0.43 |
| Residence | Rural (ref) |  |  |  |  |
|  | Urban | 1.19 | 0.79 | 1.79 | 0.41 |
| Method type | Short acting method (ref) |  |  |  |  |
|  | Long acting method | 0.32 | 0.22 | 0.48 | <0.01 |
| Type of use | Overt use (ref) |  |  |  |  |
|  | Covert use | 0.68 | 0.32 | 1.48 | 0.33 |
| Interaction | Time (months)*type of use | 1.03 | 1.00 | 1.06 | 0.02 |
| N | 1,596 |  |  |  |  |

**Appendix 4. Adjusted hazard ratios for hazard of discontinuation of contraception in Kenya (2019-2020), excluding women who switched methods**

|  |  | ADJUSTED HAZARD RATIO | | | |
| --- | --- | --- | --- | --- | --- |
|  |  | Adjusted HR | Lower CI | Upper CI | p-value |
| Age, years | 15-24 (ref) |  |  |  |  |
|  | 25-34 | 1.13 | 0.68 | 1.89 | 0.64 |
|  | 35+ | 1.56 | 0.80 | 3.07 | 0.20 |
| Highest schooling level | None/Primary (ref) |  |  |  |  |
|  | Secondary+ | 0.93 | 0.64 | 1.34 | 0.69 |
| Parity | 0-2 children (ref) |  |  |  |  |
|  | 3-4 children | 0.80 | 0.50 | 1.28 | 0.36 |
|  | 5 plus children | 0.84 | 0.45 | 1.55 | 0.57 |
| Household wealth | Lower (ref) |  |  |  |  |
|  | Middle | 0.66 | 0.44 | 1.00 | 0.05 |
|  | Highest | 0.87 | 0.49 | 1.43 | 0.59 |
| Residence | Rural (ref) |  |  |  |  |
|  | Urban | 1.11 | 0.74 | 1.65 | 0.62 |
| Method type | Short acting method (ref) |  |  |  |  |
|  | Long acting method | 0.28 | 0.19 | 0.41 | <0.01 |
| Type of use | Overt use (ref) |  |  |  |  |
|  | Covert use | 0.70 | 0.33 | 1.48 | 0.35 |
| Interaction | Time (months)*type of use | 1.03 | 1.00 | 1.06 | 0.02 |
| N | 1,488 |  |  |  |  |
